# Association of Meteorological Factors with Pediatric Intussusception in Subtropical China: A 5-Year Analysis

**DOI:** 10.1371/journal.pone.0090521

**Published:** 2014-02-28

**Authors:** Wan-liang Guo, Shu-feng Zhang, Jin-en Li, Jian Wang

**Affiliations:** 1 Radiology Department, The Children’s Hospital Affiliated to Soochow University, Suzhou, China; 2 Orthopaedics Department, Traditional Chinese Medicine Hospital of Yulin, Yulin, China; 3 General Surgery Department, The Children’s Hospital Affiliated to Soochow University, Suzhou, China; Peking Union Medical College, China

## Abstract

**Purpose:**

The aim of this study was to determine whether climate factors correlate with variations in the rate of pediatric intussusception cases presenting to the Children’s Hospital in Suzhou, China.

**Material and Methods:**

The hospital records of 5,994 pediatric cases of intussusception who had presented between Aug 2006 and Dec 2011 were retrospectively analyzed. Demographic data and air enema reduction data were collected for each case.

**Results:**

The monthly rate of new intussusception cases fluctuated throughout the year generally rising from April to September with a peak from May to July. This annual cycling of intussusception incidence was highly significant over the 5 year observation period. Poisson regression analysis showed that the monthly number of intussusception cases was associated with an increase in mean temperature per month (P = 0.0001), sum of sunshine per month (P<0.0001), precipitation per month (P<0.0001), and was marginally associated with increased mean wind speed per month (P = 0.0709).

**Conclusion:**

The incidence of intussusception in Suzhou was seasonally variable with a peak in cases presenting during hotter, sunnier, and wetter months demonstrating a positive association with certain climatic factors.

## Introduction

Intussusception is a common emergency in infants and children, and in particular in children below the age of 1 year [1], [2], [3], [4]. The majority of intussusception cases in children are primary and their cause is still unclear. Although there have been some reports about seasonal patterns in the occurrence of intussusception [5], [6], this topic remains controversial. Up until now, there have been very few studies regarding the relationship between the occurrence of intussusception and climate factors. The aim of this study was to determine whether climate factors such as temperature, sunshine, precipitation, and wind speed correlate with variations in the incidence of intussusception in Suzhou.

## Methods

This study was approved by the Institutional Review Board of the Children’s Hospital affiliated with Soochow University, which has 800 inpatient beds and is the only pediatric health care institute in Suzhou. Informed consent was signed by the parents or designated guardians of each patient. The gold standard for diagnosis of intussusception was used which included a combination of clinical assessment and imaging materials that were then standardized using major and minor criteria [7], [8]. The medical charts were retrospectively reviewed for patients under the age of 12 years who had been hospitalized with intussusception between Aug 2006 and Dec 2011 (5,994 cases). Demographic data and air enema reduction data were collected.

Meteorological data (monthly mean temperature (°C), precipitation levels, sum of sunshine (h), and mean wind speed (m/s)) were provided by the Meteorological Bureau of Suzhou, Suzhou City, China.

### Statistical Analysis

We used SAS 9.2 Proc Spectra with Fisher’s Kappa test to test the annual periodicity of the number of intussusception cases that presented to the Children’s Hospital monthly during a period from 2006 to 2011. Due to the relative rarity of intussusception cases and the non-normal distribution of new cases per month throughout each calendar year, we performed Poisson regression analysis using Proc Genmod to evaluate the relationships between the number of new intussusception cases per month (dependent variable: Y) and the following 4 climate factors (independent variables): mean temperature per month (°C), sum of sunshine per month (h), precipitation per month (mm) and mean wind speed per month (m/s). Probability values of P<0.05 were considered statistically significant.

## Results

### 3.1 Overview of Intussusception Demographic Data

The overall number of intussusception cases from Aug 2006 to Dec 2011 was 5,994. There were 3,901 (65.08%) male and 2,093 (34.92%) female patients (male to female ratio, 1.86∶1). There were 3,118 patients below the age of 1 year, 1,609 between 1–2 years of age, 564 between 2–3 years of age, and 622 patients were over 3 years old (Table 1). There were 5,560 cases (92.75%) in which reduction by air enema successfully reduced the outward size of the bowel, and 434 cases (7.25%) in which this treatment was unsuccessful and further surgical intervention was required.

**Table 1:** Distribution of intussusception cases by age groups and sex throughout the years.

	Agegroup(years)				total	Sex
	0–1	1–2	2–3	>3		male
2006 (Aug–Dec)	164	72	25	17	278	190
2007 (Jan–Dec)	530	246	94	78	948	620
2008 (Jan–Dec)	581	306	83	104	1074	683
2009 (Jan–Dec)	603	308	103	137	1151	723
2010 (Jan–Dec)	575	355	109	113	1152	726
2011 (Jan–Dec)	665	403	150	173	1391	959
total	3118	1690	564	622	5994	3901

### 3.2 Overview of the Intra-annual Intussusception Cycle

**Figure 1:**
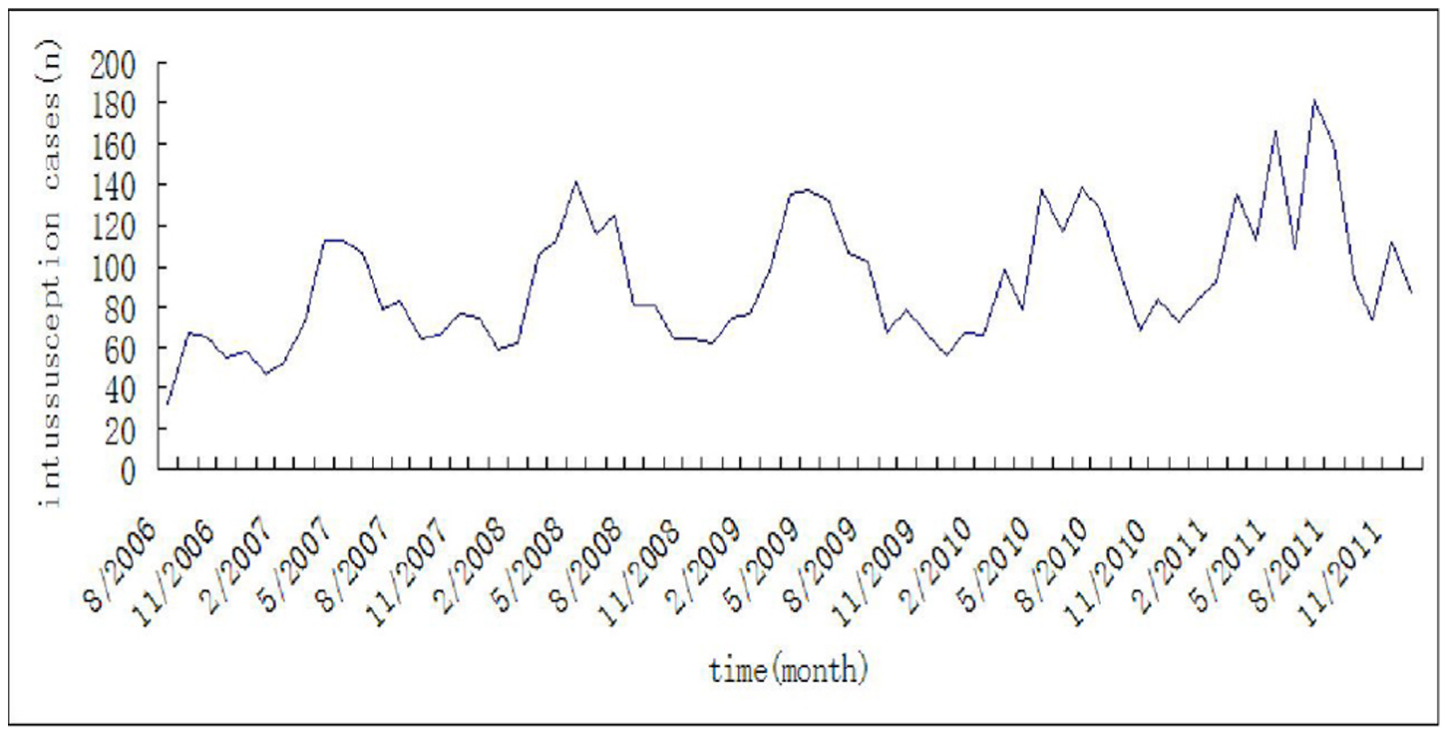
Monthly distribution of intussusception cases between Aug 2006 and Dec 2011 presenting to the Children’s Hospital of Suzhou.

The monthly rate of intussusception cases presenting to the Children’s Hospital from Aug 2006 to Dec 2011 was evaluated. While the annual rate of intussusception did not vary, the monthly rate of intussusception varied greatly throughout each year during this 5 year period, with an annual peak from May to July and a trough from October to January (Figure 1). Fisher’s Kappa test statistic of 13.81 was larger than the 5% critical value of 7.2; therefore, the null hypothesis that the monthly incidence of intussusception cases was not associated with the seasonal cycle was rejected, i.e., seasonal periodicity existed through 2006 to 2011.

### 3.3 Correlation between the Monthly Number of Intussusception Cases and Climate Variation

**Figure 2:**
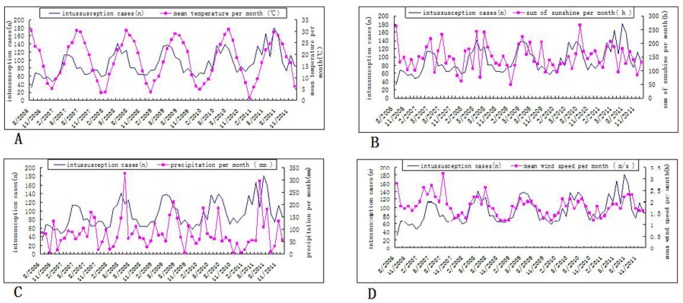
Monthly variation of climatic factors and intussusception cases from Aug 2006 to Dec 2011. A. Mean temperature per month and intussusception cases. B. Mean sum of sunshine per month and intussusception cases. C. Mean precipitation per month and intussusception cases. D. Mean wind speed per month and intussusception cases.

Mean monthly temperatures in Suzhou ranged from 0 to 33, mean monthly hours of sunshine ranged from 60 h –275 h, mean monthly precipitation ranged from 0 mm to 340 mm, and mean wind speed ranged from 1.4 m/s to 3.4 m/s. As the monthly means for each of these 4 climatic variables rose during the year, so did the mean number of new intussusception cases, and as the monthly mean value for these 4 climatic variables fell during the year, so, too, did the rate of intussusception in Suzhou Children’s Hospital (Figure 2). Poisson regression analysis of these data showed that, during the 5-year study period, the monthly incidence of intussusception cases was positively associated with the mean temperature per month (P = 0.0001), sum of sunshine per month (P<0.0001), precipitation per month (P<0.0001), and was marginally association with the mean wind speed per month (P = 0.0709) (Table 2).

**Table 2:** Poisson regression analysis of the relationship between the monthly number of intussusception cases and four climate factors.

variables	Regressioncoefficient (β)	Standard error (S_β_)	95%CI for β	χ ^2^ -value	*P* value
intercept	3.9318	0.0632	3.8079–4.0557	3868.32	<0.0001
mean temperature per month (C)	0.0081	0.0021	0.0040–0.0123	14.82	0.0001
sum of sunshine per month (h)	0.0014	0.0004	0.0007–0.0022	14.59	<0.0001
precipitation per month (mm)	0.0011	0.0002	0.0006–0.0016	20.07	<0.0001
mean wind speed per month (m/s)	0.0716	0.0405	−0.0077–0.1509	3.13	0.0709

## Discussion

The idea of seasonal patterns in the incidence of intussusception remains controversial. Chen et al. analyzed 7,541 intussusception cases in Taiwan between 1998–2007, and concluded that the mean number of monthly cases was significantly higher during the warmer months than in the cooler months [5]. Bines et al. reported that intussusception occurred particularly in summer months in tropical and subtropical regions [9]. In a survey of Hong Kong government hospitals, Nelson et al. reported 190 intussusception cases in one year and found evidence of a peak from May to July [10]. Interestingly, Sáez-Llorens et al. collected data from 476 intussusception cases in 16 centers in 11 Latin American countries and found that the occurrence of intussusception patterns varied in different geographic regions [11]. However, Ho et al. reported 952 intussusception cases in Taiwan from 1999 to 2001, and they concluded that there was no seasonal trend [12]. Enweronu-Laryea et al. reported no intussusception seasonality in a 5-year study of Ghanaian children [13]. Khumju et al. reported 72 intussusception cases over 3 years in Thailand finding that the peak of intussusception incidence for this sample was in the winter [14]. While the size and extent of the Chen study [5] is especially robust and provides support for our data, the review of the literature emphasizes that unexplained variability in the seasonal periodicity of intussusception exists. Nonetheless, a strong body of literature now suggests that variations in geographic and climatic factors might correlate with the occurrence of intussusception providing rationale for further investigation.

Here, we presented 5,994 intussusception cases over a period of more than 5 years with an age from 0–12 years. The epidemiology of intussusception in Suzhou was similar to that reported in other parts of the world [5], [6]. Eighty-three percent of patients were under 1 year of age, with 63% between 3 and 9 months. The age distribution of intussusception was similar to a previous Thai study, which found that 86% of the patients were younger than 1 year of age [14]. The sex ratio of intussusception cases has been reported to vary greatly across different regions, but all reports have indicated predominance in males. Here, we also had a higher proportion of male (65.08%) than female patients. In addition, air enema reductions were successful in 92.75% of patients, similar to what has been previously reported [7], [15], [16].

We quantified pediatric intussusception in our hospital in Suzhou during a 5 year period and we examined the association of climatic factors such as monthly mean temperatures, precipitation, sum of sunshine, and mean wind speed with the occurrence of childhood intussusception. As show in Figure 1, more intussusception cases were admitted during the summer and spring seasons in this region presenting most frequently from April to September and peaking from May to July, with temperatures of 16–30.9°C, precipitation of 55.1–326.1 mm, and sum of sunshine of 76.6–266.4 h. In contrast, intussusception cases were relatively uncommon during the cold months, with temperatures of 1.1–21.7°C, precipitation of 2.9–146.2 mm, and sum of sunshine of 48.7–205.5 h. In general, higher monthly rates of intussusception cases were associated with higher mean temperature per month, more sunshine per month, and more precipitation per month (Table 2, Figure 2).

Several limitations of the present study should be acknowledged. First, this is a retrospective study. Second, we only considered four climate variables, omitting other climate factors that may provide important information for hypothesis testing regarding the intussusception occurrence. Third, there has been a growing floating population in Suzhou in recent years; therefore, the number of children has been variable throughout each year making it difficult to correctly estimate the incidence rate of intussusception in Suzhou.

In summary, this study provides baseline data relevant to the epidemiology of intussusception in infants and children in our hospital in Suzhou. We found that there was seasonal variation of intussusception in Suzhou. Pediatric intussusception occurrence in our hospital in Suzhou peaked annually in hot months over the 5 year observation period. We found significant positive correlations between the occurrence of intussusception and climatic factors including mean temperature per month, sum of sunshine per month and precipitation per month. Our study provides reasonable evidence to educate at-risk populations during high-occurrence seasons to prevent severe complications from intussusception.
